# The Role of Early Serum Biomarkers and Clinical Rating Scales in the Prediction of Delayed Cerebral Ischaemia and Short-Term Outcome after Aneurysmal Subarachnoid Haemorrhage: Single Centre Experience

**DOI:** 10.3390/jcm12175614

**Published:** 2023-08-28

**Authors:** Małgorzata Burzyńska, Agnieszka Uryga, Jowita Woźniak, Rafał Załuski, Chiara Robba, Waldemar Goździk

**Affiliations:** 1 Department of Anaesthesiology and Intensive Care, Wroclaw Medical University, 50-367 Wroclaw, Poland; malgorzata.burzynska@umw.edu.pl (M.B.); waldemar.gozdzik@umw.edu.pl (W.G.); 2 Department of Biomedical Engineering, Faculty of Fundamental Problems of Technology, Wroclaw University of Science and Technology, 50-370 Wroclaw, Poland; 3 Department of Neurosurgery, Wroclaw Medical University, 50-367 Wroclaw, Poland; jowita.wozniak@umw.edu.pl (J.W.); rafal.zaluski@umw.edu.pl (R.Z.); 4 Anesthesia and Intensive Care, San Martino Policlinico Hospital, IRCCS for Oncology and Neurosciences, 16132 Genoa, Italy; kiarobba@gmail.com; 5 Department of Surgical Sciences and Integrated Diagnostics (DISC), University of Genoa, 16145 Genoa, Italy

**Keywords:** SAH, personalized medicine, outcome assessment

## Abstract

Considering the variety of complications that arise after aneurysmal subarachnoid haemorrhage (aSAH) and the complex pathomechanism of delayed cerebral ischaemia (DCI), the task of predicting the outcome assumes a profound complexity. Therefore, there is a need to develop early predictive and decision-making models. This study explores the effect of serum biomarkers and clinical scales on patients’ outcomes and their interrelationship with DCI and systemic complications in aSAH. This was a retrospective analysis including aSAH patients admitted to the Wroclaw University Hospital (Wrocław, Poland) from 2011 to 2020. A good outcome was defined as a modified Rankin Scale (mRS) score of 0–2. The prediction of the development of DCI and poor outcome was conducted using logistic regression as a standard model (SM) and random forest as a machine learning method (ML). A cohort of 174 aSAH patients were included in the analysis. DCI was diagnosed in 79 (45%) patients. Significant differences between patients with poor vs. good outcome were determined from their levels of albumin (31 ± 7 vs. 35 ± 5 (g/L); *p* < 0.001), D-dimer (3.0 ± 4.5 vs. 1.5 ± 2.8 (ng/mL); *p* < 0.001), procalcitonin (0.2 ± 0.4 vs. 0.1 ± 0.1 (ng/mL); *p* < 0.001), and glucose (169 ± 69 vs. 137 ± 48 (nmol/L); *p* < 0.001). SM for DCI prediction included the Apache II scale (odds ratio [OD] 1.05; 95% confidence interval [CI] 1.00–1.09) and albumin level (OD 0.88; CI 0.82–0.95). ML demonstrated that low albumin level, high Apache II scale, increased D-dimer and procalcitonin levels had the highest predictive values for DCI. The integration of clinical parameters and scales with a panel of biomarkers may effectively facilitate the stratification of aSAH patients, identifying those at high risk of secondary complications and poor outcome.

## 1. Introduction

Aneurysmal subarachnoid haemorrhage (aSAH) is a cerebrovascular disease that accounts for approximately 5–7% of all strokes. The incidence of aSAH in 2010 was estimated to be around 6.1 (4.9–7.5) cases per 100,000 persons per year, with a notable occurrence of adverse neurological outcomes and an overall mortality rate of approximately 40%. Within this mortality rate, 10–15% of patients die in the pre-hospitalization period [1,2,3]. Among those who do receive medical attention, roughly 25% die within the first 24–72 h. While advancements in care have resulted in a decrease in mortality by about 0.9% annually, long-term outcomes still remain unfavourable. In terms of long-term consequences, about 25% of patients experience cognitive impairment and exhibit focal neurological deficits [4,5,6].

**Figure 1:**
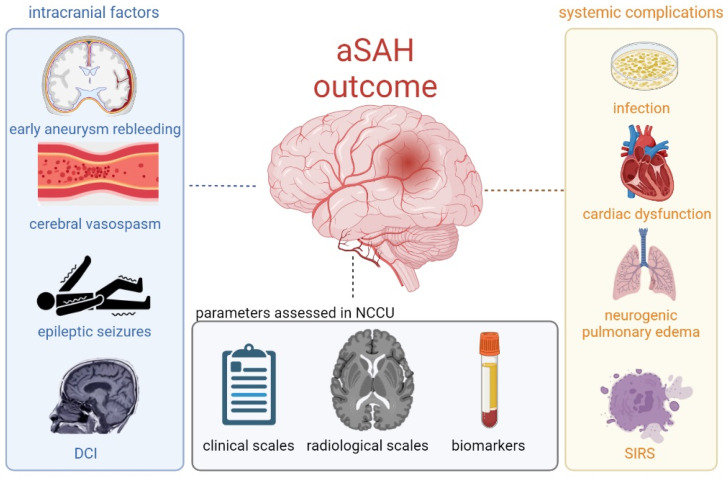
A schaematic presentation of factors that influence poor outcome after aneurysmal subarachnoid haemorrhage (aSAH). Abbreviations: NCCU, neurocritical care unit; DCI, delayed cerebral ischaemia; SIRS, systemic inflammatory response syndrome.

The primary factors associated with outcome in aSAH include early aneurysmal rebleeding, epileptic seizures, cerebral vasospasm (CV), and delayed cerebral ischaemia (DCI) [7]. Among patients who survive ruptured aneurysm repair, DCI remains the leading cause of morbidity and mortality. It occurs in approximately 30% of individuals who have initially experienced the haemorrhage, typically between 4 and 10 days after the onset of aSAH. While CV has long been recognized as the underlying pathological mechanism of DCI, current evidence suggests that the pathophysiology of DCI is considerably more intricate, involving multiple factors. It originates during the acute phase of brain injury (EBI) [7,8] and encompasses the loss of cerebral autoregulation, microcirculatory dysfunction, microthrombosis, cortical spreading depolarization, and neuroinflammation [9,10] (Figure 1).

Serum biomarkers serve as valuable indicators of normal biological processes, pathological processes, or the body’s response to exposure or intervention [11]. Recent evidence supports the involvement of neuroinflammation, which is initiated during EBI, in the pathogenesis of cerebral cell damage and the onset of CV after aSAH [12]. Erythrocyte degradation products present in the subarachnoid space trigger an inflammatory response from the resident central nervous system (CNS) gliomas directly via TLR4 receptor signalling. Reactive microglia contribute to the production of inflammatory cytokines, vasoconstriction, and neuronal apoptosis. Simultaneously, the up-regulation of cerebral vascular endothelial adhesion molecules enables peripheral inflammatory cells to reach the subarachnoid space. Neutrophils and macrophages initiate the phagocytosis of complex haemoglobin/haptoglobin, leading to their entrapment and degranulation in the subarachnoid space. This results in the release of inflammatory cytokines and vasoactive factors such as endothelin, triggering oxidative stress and contributing to brain inflammation, aseptic meningitis, and cerebral vasoconstriction. The severity of this process is amplified when there is a large amount of blood in the subarachnoid space, overwhelming the resident CNS macrophages (microglia) due to extensive haemolysis [13,14]. Furthermore, various indirect markers of inflammation, including lactate levels, leukocyte counts, C-reactive protein levels, and procalcitonin levels, have been investigated for their potential as predictors of CV development, DCI, and outcome. While elevated levels of these markers may be linked to the occurrence of CV and DCI, it is important to highlight that they primarily impact poor outcomes, indicating that these markers may serve as warning signs for the likelihood of systemic complications [15,16].

Given the complex pathomechanism of DCI and the range of complications following aSAH, predicting outcomes can be challenging. Therefore, there is a need to develop early predictive and decision-making models. This study aimed to investigate the impact of serum biomarkers, radiological, and clinical scales on poor outcomes and their interrelationship with DCI and systemic complications in aSAH. This was achieved via a retrospective analysis of patients admitted to our clinical centre in Poland, Central Europe, over a span of nearly ten years.

## 2. Materials and Methods

### 2.1. Ethics Approval and Consent to Participate

The study conforms to the Declaration of Helsinki, and the research protocol was approved by the Bioethics Committee at the Medical University in Wroclaw (KB—620/2020), which waived the requirement for informed consent based on the study’s retrospective design.

### 2.2. Study Population

This retrospective single-centre study included aSAH patients admitted to the Intensive Care Unit of Wroclaw University Hospital (Wrocław, Poland) from January 2011 to December 2020. Inclusion criteria were 18 years of age or older; admission to the hospital within 48 h of the onset of clinical symptoms due to SAH from a ruptured aneurysm confirmed via computed tomography (CT), four-vessel digital subtraction angiography, or conventional angiography; and aneurysm clipping or coiling during the first 48 h following admission. Exclusion criteria were SAH related to mycotic aneurysm, arteriovenous malformation, or trauma. Additionally, patients who were previously diagnosed with stroke or brain injury with chronic changes in CT, patients admitted with a diagnosis of brain death, and patients whose biomarker levels were related to diseases other than aSAH (cancer, severe kidney or liver disease, active or chronic inflammatory diseases) were excluded. Patients were treated based on standard SAH guidelines including early diagnosis, surgical clipping or coiling, and admission to the intensive care unit [17,18,19]. The choice of surgical treatment of the aneurysm was determined following a multidisciplinary discussion including vascular neurosurgeons and an interventional neuroradiologist depending on the location, size, and morphological features of the aneurysm; the presence of a mass effect caused by the aneurysm and/or an associated haematoma; the presence of multiple aneurysms; and the patient’s neurological and general medical condition. The decision to perform cerebrospinal fluid drainage and/or decompressive craniectomy was made at various stages of the hospital stay. The treatment algorithm included nimodipine and symptomatic treatment such as the maintenance of adequate blood pressure, mechanical respiratory support, proper fluid infusion, prevention of stress ulcers, appropriate anti-infection therapy, and anti-oedema therapy to reduce intracranial pressure when necessary [19,20,21]. In patients with CV or DCI, induced hypertension was used in the first instance to improve cerebral blood flow. A control CT scan was performed on all patients within 24 h following the coiling or clipping of the aneurysm to exclude or confirm any ischemic lesions directly related to the procedure. Further follow-up CT scans were performed during the hospital stay depending on clinical indications. Neurological deficits and deterioration in the level of consciousness were investigated daily via physical examination.

### 2.3. Data Collection

Patients’ clinical status was assessed using the Glasgow Coma Scale (GCS) [22], Full Outline of UnResponsiveness scale (FOUR) [23], Acute Physiology and Chronic Health Evaluation II scale (Apache II) score [24] within the first 24 h, Hunt and Hess grade (H-H) [25], and World Federation of Neurosurgical Societies (WFNS) scale [26]. The severity of subarachnoid bleeding was classified according to the modified Fisher (mFisher) score [27]. Patients’ non-contrast CT scans were also examined, and the 10 cisterns and the 4 ventricles were assessed to provide the total Hijdra score [28]. The SubarachnoidHemorrhage Early Brain Edema Score (SEBES) was used for the estimation of the level of cerebral oedema [29], where a SEBES of 3–4 was classified as high. We categorized the aneurysm by size as small (≤12 mm), medium (13–24 mm), or large (≥25 mm).

### 2.4. Selection of Serum Biomarkers

Laboratory test results were collected upon admission (within 24 h after initial haemorrhage) and before treatment of aneurysms and included serum biomarkers, such as white blood cells (WBC), high-sensitivity C-reactive protein (CRP), D-dimer, procalcitonin (PCT), blood lactate (LAC), serum albumin, blood glucose, and CRP/albumin ratio. In addition, the role of dysglycaemia, electrolyte abnormalities, and serum fibrin breakdown products were investigated. The analyses were performed at the Academic Centre for Laboratory Diagnostics according to the laboratory standards.

### 2.5. Definitions and Endpoints

EBI was defined as disorders based on clinical symptoms, neuroimaging findings, and metabolic variables upon admission to the hospital [30]. A DCI was defined as the development of new focal or global (2-point decrease on GCS) neurological impairment lasting for at least one hour (delayed ischemic neurologic deficit, DIND), and/or cerebral infarction on follow-up CT scan (which is attributable to ischaemia, not apparent between 24 and 48 h after early aneurysm occlusion, and not attributable to other causes) [31,32]. Based on the foregoing definition and method of diagnosing DCI, patients were divided into three groups (see Table 1) [32,33,34,35]. Group one consisted of patients diagnosed with DCI based on clinical examination only (DIND). Group two comprised a combination of clinical and radiological examination (DIND and new cerebral infarction). Group three was defined based on radiological examination only (new cerebral infarction on the follow-up CT scan). The exclusion criteria for DCI were as follows: seizures, acute hydrocephalus, systemic disorders, association with sedative drugs, or causal relationship related to neurosurgical procedure within 24 h. In our study, all three categories were analysed together. Routine monitoring for detecting CV included serial transcranial Doppler ultrasonography (TCD) measurements with a 2 MHz probe (Doppler BoxX, DWL Compumedics Germany GmbH, Singed, Germany). Clinical CV was defined as mean cerebral blood flow velocity (CBFV) in the middle cerebral artery exceeding 120 cm/s or a daily increase of CBFV of 20% when measured using TCD. The anterior circulation included internal carotid arteries (ICA), anterior cerebral arteries (ACA), anterior communicating arteries (ACoA), and middle cerebral arteries (MCA). Systemic inflammatory response syndrome (SIRS) was defined according to the Centers for Disease Control and Prevention criteria as the presence of two or more of the following: heart rate > 90 bpm, respiratory rate of >20 breaths/min, temperature <36 °C or >38 °C, and WBC count <4000 or >12,000/mm^3^. Neurogenic pulmonary oedema was defined as clinical manifestations of pulmonary involvement bilateral infiltrate in the chest X-ray, PaO_2_/FIO_2_ < 200, no evidence of left atrial hypertension, and absence of other common causes of acute respiratory distress. Cardiac complications were diagnosed based on elevated cardiac enzymes, changes to the electrocardiogram, and cardiac dysfunction confirmed via transthoracic echocardiography. The outcome was assessed at discharge from the hospital using the modified Rankin Scale (mRS) [36]. A good outcome was defined as 0–2 on the mRS and a poor one as 3–6 on the mRS.

### 2.6. Statistical Methods

The hypothesis of normality was rejected for most of the analysed variables; therefore, nonparametric methods were used. The level of significance was set as α = 0.05. Frequencies were compared using the χ^2^ test. The distributions of quantitative variables were compared using the U Mann–Whitney test. To determine the discriminative value of the biomarkers, receiver operating curves (ROC) were utilized, and the threshold was determined using the Youden index. The Kaplan–Meier plot and Cox proportional hazards model with F Cox statistics were used to assess differences in mortality in patients with disturbed and normal biomarkers. In the analysis of survival, patients who were alive were classified as censored observations, and the survival time consisted of the total time of hospitalization (including time spent in the ICU and in the neurosurgical ward). As predictors, the following variables were introduced into the models: covariates (age, gender, BMI), serum biomarkers, and all clinical scales. The standard model (SM) was a multivariate logistic regression analysis, performed using the backward selection method. The results were reported as odds ratios, and the models were assessed using χ^2^ statistic and Akaike Information Criterion (AIC). The collinearity of the variables and significance in the univariant analysis were tested. The machine learning (ML) method—random forest—was used as an additional approach for prediction modelling. The dataset was split into training and test sets (75%:25%). Stratified 10-fold and 5-fold cross-validation approaches were used to tune the model measures (hyperparameters) for poor outcome and DCI, respectively. The random forest model was developed using the scikitlearn libraries available in the Python programming language (v3.10.6). The SHAP (SHapley Additive exPlanations) method was used to determine which variables were the strongest predictors of poor outcome. Statistical analyses were performed using STATISTICA v.13 (Tibco, Palo Alto, CA, USA). Descriptive statistics are presented as median and interquartile range.

## 3. Results

### 3.1. Baseline Characteristics and Clinical Data

**Figure 2:**
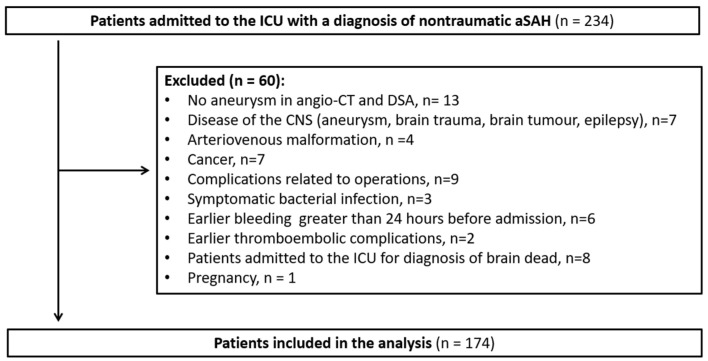
Flow chart. Abbreviations: aSAH, aneurysmal subarachnoid haemorrhage; angio-CT, computed tomography angiography; DSA, digital subtraction angiography; CNS, central nervous system; ICU, intensive care unit.

Between January 2011 and December 2020, 234 patients with non-traumatic SAH were admitted to the Intensive Care Unit of Wroclaw University Hospital and were assessed for inclusion. Of these, 174 were included in this study (Figure 2). Patients’ characteristics according to good and poor outcome are shown in Table 1. The median age was 56 years ± 22, and 105 patients (60%) were women. Among the patients chosen for inclusion, 49% of patients had a history of hypertension, while 43% had a history of smoking. In 169 patients (97%), the ruptured aneurysm was in their anterior circulation. Multiple aneurysms were recorded in 54 cases (31%). Early CV in the first computed tomography angiography (CTA) was recorded in 21 cases (13%). Of those studied, 102 patients (59%) underwent surgical clipping, and 72 patients (41%) were treated with endovascular coil embolization; 93 (55%) patients met the pre-set criteria for CV on TCD or angiography.

Based on DIND and cerebral infarction on follow-up CT, DCI was diagnosed in 66 (38%) of the patients; however, based on DIND alone, DCI occurred in 13 (7%) patients. Ultimately, DCI was defined in 79 patients, which is 45% of the total group; see Table 1. SIRS occurred in 62 patients, and 24 developed multiple organ failure. The most prevalent nosocomial infections were pneumonia (n = 60, 34%) and urinary tract infection (n = 38, 22%). Meningitis/ventriculitis was diagnosed in seven patients (4%) and was associated with the presence of intraventricular haemorrhage and external ventricular drainage. In the study cohort, 43 (24%) of patients died and 46 (26%) survived with poor outcomes. Hospital mortality occurred after a mean of 20 ± 19 days (min: 4; max: 76) from the date of aSAH development. The main cause of death was irreversible brain damage (n = 24/43; 56%), followed by multiple organ failure (n = 13/43; 30%) and circulatory failure (n = 6/43; 14%).

### 3.2. Comparison between Patients with Poor Outcome vs. Good Outcome

In comparison with patients with good outcomes, those with poor outcomes were older (61 ± 24 vs. 52 ± 19 years; *p* = 0.005), had higher H-H (4 ± 2 vs. 3 ± 1; *p* < 0.001), WFNS (5 ± 1 vs. 2 ± 2; *p* < 0.001), and mFisher scores (4 vs. 3 ± 2; *p* < 0.001). Moreover, patients with poor outcomes had higher Hijdra sum scores (23 ± 17 vs. 12 ± 21; *p* < 0.001) and SEBESs (3 ± 3 vs. 1 ± 3; *p* < 0.001) in comparison to those with good outcomes. Significant differences were found in clinical scales between patients with poor vs. good outcome (Table 1). ROC curve analysis showed that all covariates were significant predictors of poor outcome (Figure S1A). Systemic complications occurred more frequently in patients with poor outcome than in ones with good outcome, see Table 1.

### 3.3. Correlation between Serum Biomarkers and Poor Outcome

**Figure 3:**
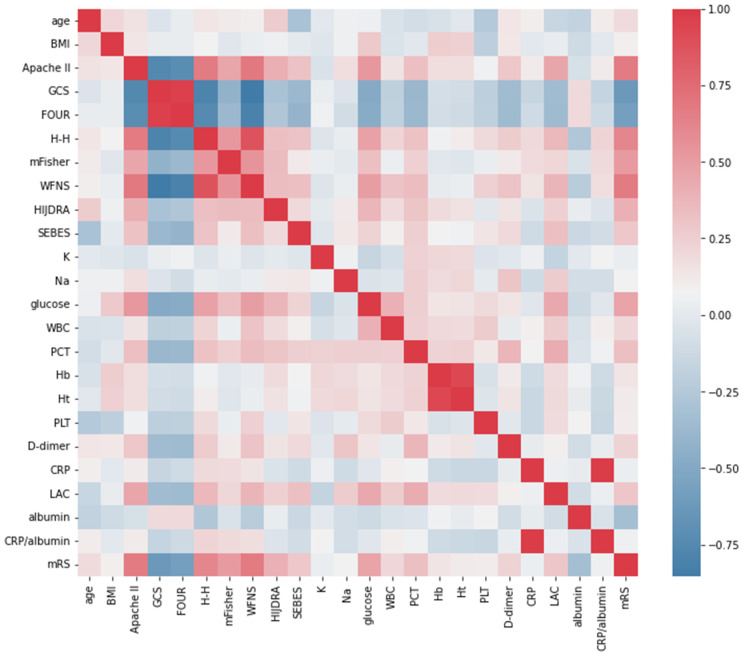
Matrix of Spearman’s correlation between covariate (age, body mass index (BMI)), clinical scales, biomarkers, and outcome assessed via modified Rankin Scale (mRS) in the total group of aneurysmal subarachnoid haemorrhage (aSAH) patients. Darker red signifies a positive correlation while darker blue signifies a negative correlation among all aSAH patients. Abbreviations: Apache II, Acute Physiology and Chronic Health Evaluation; GCS, Glasgow Coma Scale; FOUR, Full Outline of UnResponsiveness scale; H-H, Hunt and Hess scale; mFisher, modified Fisher scale; WFNS grade, World Federation of Neurosurgical Societies grade; SEBES, Subarachnoid Haemorrhage Early Brain Edema Score; K, potassium; Na, sodium; WBC, white blood cells; PCT, procalcitonin; Hb, haemoglobin; Ht, haematocrit; CRP, C-reactive protein; PLT, platelets; LAC, serum lactate level.

The matrix of Spearman’s correlation between biomarkers, covariates (age, BMI), and clinical scales is presented in Figure 3.

All serum biomarkers at admission were elevated in patients with poor outcome; see Table 2. Significant differences between patients with poor vs. good outcome were found in levels of CRP/albumin ratio (0.2 ± 0.5 vs. 0.1 ± 0.2; *p* = 0.040), D-dimer (3.0 ± 4.5 vs. 1.5 ± 2.8 (ng/mL); *p* < 0.001), PCT (0.2 ± 0.4 vs. 0.1 ± 0.1 (ng/mL); *p* < 0.001), LAC (1.6 ± 1.6 vs. 1.0 ± 0.8 (nmol/L); *p* = 0.001), and glucose (169 ± 69 vs. 137 ± 48 (nmol/L); *p* < 0.001).

ROC curve analysis showed that all biomarkers analyses were significant predictors of poor outcome (see Table 3 and Figure S1B). Moreover, we found that increased levels of D-dimer, glucose, and PCT, as well as decreased levels of albumin, significantly lowered the probability of survival (Table S1 and Figure S2).

### 3.4. SEBES vs. DCI Development and Outcome

Patients with high SEBES were younger (51 ± 21 vs. 61 ± 18, *p* < 0.001) and had worse initial clinical conditions in comparison with patients with low SEBES: GCS (7 ± 8 vs. 13 ± 11, *p* < 0.001), Apache II (20 ± 13 vs. 13 ± 11, *p* < 0.001), and H-H (4 ± 2 vs. 3 ± 2, *p* < 0.001). Significant differences between patients with high vs. low SEBES were found in selected biomarkers: PCT (0.15 ± 0.44 vs. 0.07 ± 0.14 (ng/mL), *p* < 0.001), LAC (1.5 ± 1.9 vs. 1.1 ± 1.0 (mmol/L), *p* = 0.021), and glucose (166 ± 68 vs. 148 ± 57 (mmol/L), *p* = 0.015). Moreover, patients with high SEBES presented more systemic complications, such as pneumonia (*p* = 0.002), SIRS (*p* = 0.005), and cardiac complications (*p* < 0.001). They also had worse outcomes (mRS 4 ± 4) in comparison with the low-SEBES group (mRS 2 ± 4), *p* < 0.001. Details of the characteristics of patients in the low- and high-SEBES subgroups, respectively, are presented in Table S2.

### 3.5. Prediction of Poor Outcome Using SM and ML Approach

**Figure 4:**
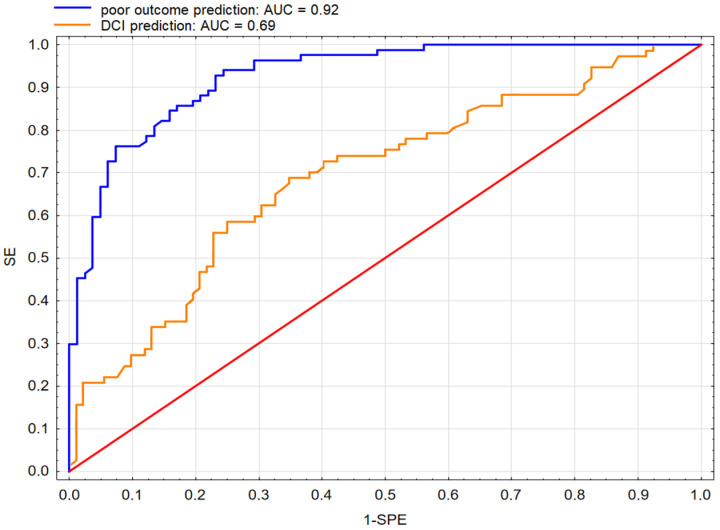
The receiver operating characteristic (ROC) curve for the logistic regression model predicts poor outcome (**blue**) and delayed cerebral ischaemia (DCI) (**orange**). The area under the curve (AUC) for the models is presented in the figure. Abbreviations: SE, sensitivity; SPE, specificity.

The SM (Table 4) correctly classified 84% of cases and was statistically significant: χ^2^ = 99.16; *p* < 0.001, AIC = 118.36. Apache II score, mFisher score, SEBES, and albumin levels were included in the final model. We found that an increase of one point on the Apache II scale caused 20% more risk of a poor outcome, and a single change in mFisher caused a three times higher risk of poor outcome. A single increase in SEBES indicated a 38% greater chance of a poor outcome. In addition, a decrease in albumin level increased the risk of a poor outcome by about 17%. The area under the curve (AUC) for this model was 0.92 (Figure 4). Other metrics of model evaluation were as follows: accuracy: 83%, sensitivity: 83%, specificity: 82%, positive predictive value: 84%, and negative predictive value: 82%.

**Figure 5:**
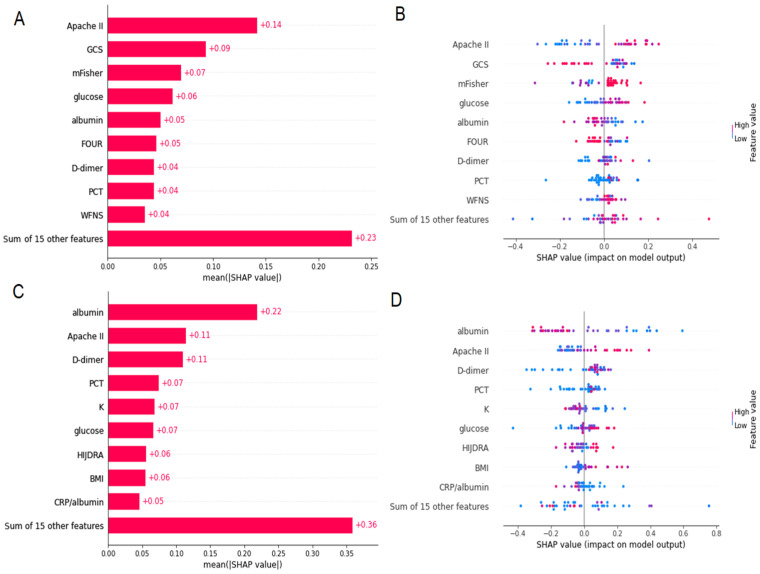
Prediction of poor outcome (**A**,**B**) and delayed cerebral ischaemia (DCI) (**C**,**D**) after subarachnoid haemorrhage (aSAH) using machine learning method. Variables in the machine learning approach were ranked using the random forest model to identify the relative importance of variables as predictors (see panels (**A**,**C**)). Factors’ importance and contribution plot ranked using the random forest model to identify the relative importance of variables (see panels (**B**,**D**)). Abbreviations: Apache II, Acute Physiology and Chronic Health Evaluation; GCS, Glasgow Coma Scale; mFisher, modified Fisher scale; FOUR, Full Outline of UnResponsiveness scale; PCT, procalcitonin; WFNS grade, World Federation of Neurosurgical Societies grade; K, potassium; BMI, body mass index; CRP, C-reactive protein.

The ML predicted poor outcome with an accuracy of 79%, sensitivity of 90%, specificity of 69%, positive predictive value of 74%, and negative predictive value of 87%. A confusion matrix is presented in Figure S3. The SHAP value demonstrated that Apache II, GCS, mFisher, and glucose levels had the highest predictive values for poor outcome (Figure 5A). High Apache II and mFisher scores, elevated glucose levels, and low GCS scores increased the probability of a poor outcome (Figure 5C).

### 3.6. Prediction of DCI Using SM and ML Approach

The SM (Table 4) correctly classified 66% of cases and was statistically significant: χ^2^ = 21.29; *p* < 0.001, AIC = 218.35. Of the factors included in the model, the Apache II scale and albumin levels were included in the final model. A decrease in albumin level increased the risk of DCI by about 12%. We found that every single change in the Apache II scale caused a 5% greater chance of DCI. The AUC for this model was 0.69 ± 0.04 (Figure 4). Other metrics were as follows: accuracy of 68%, sensitivity of 69%, specificity of 68%, positive predictive value of 66%, and negative predictive value of 71%.

The ML algorithm-predicted DCI had the following accuracy: 65%, sensitivity: 69%, specificity: 62%, positive predictive value: 58%, and negative predictive value: 73%. A confusion matrix is presented in Figure S3. The SHAP value demonstrated that albumin level, Apache II score, and D-dimer and PCT level had the highest predictive values for DCI (Figure 5B). Low albumin, high Apache II, and elevated levels of PCT and D-dimer increased the probability of DCI (Figure 5D).

## 4. Discussion

In this study, we aimed to assess the prognostic value of combining clinical and radiological scales with serum biomarkers in patients with aSAH. We utilized both classical statistical methods and a machine learning approach to investigate the interrelationship between predictors of poor outcome, with the goal of effectively stratifying patients at higher risk of secondary complications and poor prognosis. Our findings demonstrated that serum biomarkers were correlated with patients’ initial clinical condition, a higher incidence of complications, and poor outcomes. In our study group, which consisted of patients with poor outcomes, we diagnosed a higher number of individuals with both neurological and systemic complications. This suggests that beyond focusing solely on neurological complications, early recognition of systemic complications with potential adverse effects may contribute to improving the outcomes of patients with aSAH.

We observed the strongest association between the development of DCI, as diagnosed by the full 2010 consensus definition [32] and the levels of albumin during the acute phase, D-dimer levels, and Apache II score. However, it remains unclear whether these observed changes solely predict the development of DCI, susceptibility to other complications, or both. The other scales used, such as Hunt and Hess grade, Fisher scale score, aneurysm location, treatment modality, and gender, although associated with treatment outcome, were not strongly associated with DCI. Although a systematic review showed a relationship between the Hijdra sum score, Fisher score, and modified Fisher score with DCI [37], another study strictly adhering to the DCI definition demonstrated no association between DCI and the degrees scored on the Hunt and Hess scale and Fisher scale [38]. This finding is consistent with our observations.

The perception of aSAH patients has evolved in parallel with the widespread use of mixed scales in various combinations. Examples of these multidimensional models include HAIR [39], VASOGRADE [40], HATCH [41], or EDCI [42] scales. However, it is important to note that these scales have their limitations and exhibit significant heterogeneity within study groups, particularly during the assessment phase [43]. In a recent review which assessed the methodological quality of multivariate prognostic models [43], only the SAHIT scale [44] demonstrated clear discrimination in predicting probabilities of mortality and functional outcomes at three months. Nevertheless, it is worth mentioning that the SAHIT scale does not incorporate laboratory biomarkers that could indicate the development of both neurological and systemic complications, which can significantly impact treatment outcomes.

Previous studies have consistently reported a relationship between Fisher radiological score and the prognosis of aSAH patients [42,45,46]. Another radiological scale tool, the SEBES scale, which assesses cerebral oedema, has also been investigated for its predictive value in identifying secondary complications, including an increased risk of developing DCI. Ahn et al. [29] conducted a study examining the dynamics of cerebral oedema changes based on SEBES classification along with the simultaneous assessment of circulating cytokines. Their findings demonstrated a correlation between persistent cerebral oedema and the dynamics of markers from the acute to subacute phase. The study revealed that patients with persistently poor or worsened SEBESs tended to be younger and had higher WFNS scores, a higher incidence of DCI, and poorer functional outcomes at three months after SAH. Furthermore, it was observed that patients with persistent cerebral oedema had elevated serum levels of eotaxin and inflammatory and regulatory cytokines and strong collinearity between these markers and the severity of SAH [29]. Similarly, in our study, although we assessed oedema at the first CT scan, we observed that patients with high SEBESs (3–4) were younger and more likely to have experienced loss of consciousness at onset. Additionally, they had poor initial results on the selected clinical and radiological scales as well as abnormal levels of biomarkers. We also found that these patients had longer stays in the ICU, experienced more neurological and systemic events, and had worse short-term outcomes. In our study, a scale significant for both DCI and poor outcome prediction was Apache II scale. Its usefulness in predicting treatment outcomes has been investigated in patients with aSAH [47,48]. The Apache II scale includes age and the Glasgow Coma Scale (GCS), which have both been identified as independent predictors of poor prognosis in patients with aSAH.

The relationship between systemic inflammation and aSAH has been extensively studied in previous research [49,50,51,52]. Inflammation is recognized as a direct contributor to neurological injury following aSAH and a potential causative factor for secondary complications such as DCI and post-SAH vasospasm [53,54,55,56]. In addition to inflammatory cytokines in cerebrospinal fluid and plasma, other systemic inflammatory markers including PCT, CRP, leukocyte, and LAC levels have been also evaluated [53,57,58,59]. Although the exact pathophysiological mechanism and causal associations between acute-phase response biomarkers and neurological outcome in patients with aSAH are not yet fully understood, their elevation may reflect the severity of the early inflammatory response triggered by aneurysm rupture, enabling the early identification of patients at risk of deterioration [50]. In our predictive models, although changes in glucose and albumin levels were the most significant among the biochemical markers, D-dimer, PCT, and LAC levels also significantly influenced the probability of survival. These physiological variables consistently accompanied poor performance in previously published studies [60,61,62,63]. Earlier studies reported that low albumin levels are associated with increased morbidity and mortality in aSAH patients [64,65,66]. Hypoalbuminemia can occur due to reduced synthesis, increased loss, or altered distribution in extracellular and intracellular spaces. In critical diseases, there is a shift in albumin distribution, as well as changes in its synthesis rate and degradation. During the early stage of the disease, albumin concentration decreases and does not increase until the recovery phase. The exact mechanism underlying hypoalbuminemia in aSAH is not fully understood, but it is believed to be one of the key markers of a complex acute systemic inflammatory response, similar to the acute phase observed in other critical illnesses [67]. In our study, we observed that albumin level upon admission was a strong predictor of both poor outcome and the development of DCI. These findings highlight the significance of albumin as an important component of the inflammatory response and its potential role in assessing disease severity and prognosis in aSAH patients.

Hyperglycaemia is commonly observed in patients with aSAH and has been associated with more severe haemorrhages and increased risk of complications during hospitalization. Furthermore, it has been independently correlated with higher mortality rates and poorer overall outcomes [68,69,70]. The presence of high glucose levels at admission is often attributed to early brain damage, and one of the underlying mechanisms is the stress response triggered by the activation of the autonomic sympathetic nervous system. This response leads to increased circulating levels of catecholamines and cortisol, which subsequently induce hyperglycaemia and insulin resistance [71,72]. In the study by Frontera et al. [73], independent indicators of high daily glucose burden were poor Hunt–Hess grade and elevated Apache II score. These findings underscore the importance of considering these clinical factors when evaluating glucose levels and their impact on patient outcomes in aSAH.

In addition to neurological (intracranial) complications that significantly impact outcomes in aSAH patients, our cohort also experienced systemic complications, including systemic inflammatory response syndrome (SIRS), pneumonia, urinary tract infections, and multiple organ failure. Importantly, these systemic complications were associated with an unfavourable outcome in our study, which aligns with previous research highlighting the detrimental effects of hospital-acquired infections following aSAH [74,75,76]. By addressing and managing these systemic complications in a timely manner, clinicians may potentially improve overall outcomes and prognosis for patients with aSAH.

### Limitations

This study has several limitations that should be acknowledged. Firstly, it is important to note that this was a retrospective, single-centre, observational study, which may limit the generalizability of the findings to other settings or populations. The observations and results presented here are specific to the clinical experience and patient population at our hospital. Another limitation is the lack of external validation using an independent cohort to confirm the robustness and reliability of the findings. Additionally, the study did not evaluate the dynamic changes in the monitored biochemical parameters over time. Furthermore, the evaluation of the modified Rankin Scale (mRS) as an outcome measure was performed at hospital discharge, which may introduce bias due to variations in hospitalization duration among patients with different outcomes. Future studies should consider longer-term follow-up assessments to provide a more comprehensive evaluation of patient outcomes. Regarding the ML model used in this study, it is important to acknowledge its limitations. Overfitting can occur when the number of trees is limited, and the model’s efficiency may be reduced when some trees have high complexity. Further optimization and refinement of the ML model are necessary to address these limitations and enhance its performance. Lastly, the study sample size was relatively small, which may limit the internal validity and statistical power of the proposed models. Additionally, the models were not compared or confronted with existing models, and the net reclassification index (e.g., SAHIT, SAFIRE models) was not evaluated. Future studies with larger sample sizes and comparative analyses are needed to further assess the internal validity and comparative performance of the proposed models.

## 5. Conclusions

The integration of clinical scales with a panel of serum biomarkers has shown promise in stratifying aSAH patients and identifying those at high risk of secondary complications and poor outcomes. We suggest that the tested biomarkers should be routinely performed for such patients. The findings of this study need to be further validated and confirmed in larger, multi-centre studies.

## Figures and Tables

**Table 1:** Baseline characteristics of the cohort of patients with aSAH versus outcome in modified Rankin Scale (mRS). Data are presented as median ± interquartile range or number of subjects (%).

Characteristic	Total N = 174	mRS 0–2 n = 85	mRS 3–6 n = 89	*p*
Age	56 ± 22	52 ± 19	61 ± 24	**0.005**
Female	105 (60%)	53 (62%)	52 (58%)	0.597
BMI	25.8 ± 6.4	25.7 ±5.8	26.3 ± 6.3	0.404
APACHE II	15 ± 12	11 ±6	22 ± 10	**<0.001**
GCS	11 ± 9	14 ±3	6 ± 7	**<0.001**
FOUR Score	14 ± 9	16 ±2	8 ± 9	**<0.001**
Comorbidities				
Hypertension	85 (49%)	43 (51%)	42 (47%)	0.654
Current smoker	75 (43%)	36 (42%)	39 (44%)	0.845
Diabetes mellitus, n (%)	10 (6%)	4 (5%)	6 (7%)	0.402
Other	15 (9%)	10 (12%)	5 (5%)	0.120
Aneurysm				
ICA	46 (26%)	22 (26%)	24 (27%)	0.871
MCA	48 (28%)	22 (26%)	26 (29%)	0.623
ACA	12 (7%)	9 (11%)	3 (3%)	0.060
ACoA	49 (28%)	23 (27%)	26 (29%)	0.752
Posterior	15 (9%)	6 (7%)	9 (10%)	0.329
Size: small	145 (83)	75 (88%)	70 (79%)	0.112
Size: medium	26 (15%)	9 (11%)	17 (19%)	0.092
Size: large	3 (2%)	1 (1%)	2 (2%)	0.570
Multiple aneurysms	54 (31%)	26 (31%)	28 (31%)	0.901
Clinical assessment				
H-H	3 ± 3	3 ± 1	4 ± 2	**<0.001**
Grade I–III	92 (53%)	67 (79%)	25 (27%)	**<0.001**
Grade IV–V	82 (47%)	18 (21%)	64 (73%)	
mFisher	4 ± 1	3 ± 2	4	**<0.001**
HIJDRA	18 ± 20	12 ± 21	23 ± 17	**<0.001**
SEBES	2 ± 4	1 ± 3	3 ± 3	**<0.001**
Grade 0–2	100 (57%)	62 (73%)	38 (43%)	**<0.001**
Grade 3–4	74 (43%)	23 (27%)	51 (57%)	
WFNS	4 ± 3	2 ± 2	5 ± 1	**<0.001**
Grade I–III	87 (50%)	65 (76%)	22 (25%)	**<0.001**
Grade IV–V	87 (50%)	20 (24%)	67 (75%)	
CV in CTA	21 (13%)	10 (12%)	11 (14%)	0.792
Aneurysm treatment				
Clipping	102 (59%)	49 (58%)	53 (60%)	0.603
Coiling	72 (41%)	36 (42%)	36 (40%)	0.616
EVD	75 (43%)	26 (31%)	49 (55%)	**0.001**
Decompressive craniectomy	36 (21%)	5 (6%)	31 (36%)	**<0.001**
Outcome				
ICU stay [days]	9 ± 12	6 ± 5	13 ± 15	**<0.001**
Hospital stay [days]	19 ± 20	18 ±10	22 ± 43	**0.039**
Dead	43 (24%)	0	43 (46%)	**<0.001**
Neurologic complications				
Cerebral infarction on follow-up CT	66 (38%)	16 (19%)	50 (56%)	**<0.001**
DCI	79 (45%)	24 (28%)	55 (62%)	**<0.001**
CV in TCD	93 (55%)	44 (52%)	49 (58%)	0.491
Criteria for DCI diagnosis				
A decline in GCS score and focal deficit	13 (16%)	8 (33%)	5 (9%)	
A decline in GCS score, focal deficit, and new infarction	54 (69%)	12 (50%)	42 (76%)	
New infarction alone	12 (15%)	4 (17%)	8 (15%)	
Systemic complications				
Pneumonia	60 (35%)	12 (14%)	48 (54%)	**<0.001**
Inflammation of the urinary tract	38 (22%)	8 (9%)	30 (34%)	**<0.001**
Cardiac complications	18 (10%)	4 (5%)	14 (16%)	**0.015**
SIRS	62 (36%)	11 (13%)	51 (57%)	**<0.001**
Meningitis/ventriculitis	7 (4%)	1 (1%)	6 (7%)	0.067
MOF	24 (14%)	1 (1%)	23 (26%)	**<0.001**
Neurogenic pulmonary oedema	46 (26%)	13 (15%)	33 (37%)	**0.001**
Days on MV	7 ± 7	2 ± 4	11 ± 7	**<0.001**

Abbreviations: ACA, anterior cerebral artery; ACoA, anterior communicating artery; APACHE, Acute Physiology and Chronic Health Evaluation; BMI, body mass index; CT, computed tomography; CTA, computed tomography angiography; CV, cerebral vasospasm; DCI, delayed cerebral ischaemia; EVD, external ventricular drainage; GCS, Glasgow Coma Scale; FOUR, Full Outline of UnResponsiveness scale; H-H, Hunt and Hess scale; ICA, internal carotid artery; ICU, intensive care unit; MCA, middle cerebral artery; mFisher, modified Fisher scale; MV, mechanical ventilation; MOF, multiple organ failure; SEBES, Subarachnoid Haemorrhage Early Brain Edema Score; SIRS, systemic inflammatory response syndrome; TCD, transcranial Doppler ultrasonography; WFNS grade, World Federation of Neurosurgical Societies grade; *p*, *p*-value; significant differences are marked in bold.

**Table 2:** Biomarker characteristics of the whole cohort of patients with aSAH versus outcome on modified Rankin Scale (mRS). Data are presented as median ± interquartile range or number of subjects (%). The normal range is presented in brackets.

Characteristic	Total N = 174	mRS 0–2 n = 85	mRS 3–6 n = 89	*p*
CRP [mg/L] (0–5)	6 ± 102	4 ± 95	8 ± 104	0.239
CRP/albumin [a.u]	0.1 ± 0.4	0.1 ± 0.2	0.2 ± 0.5	**0.040**
D-dimer [ng/mL] (0–0.5)	2.2 ± 3.4	1.5 ± 2.8	3.0 ± 4.5	**<0.001**
WBC [G/L] (4–10 × 10^3^)	13.5 ± 6.7	12.9 ± 5.1	13.8	0.104
PCT [ng/mL] (0–0.05)	0.1 ± 0.3	0.1 ± 0.1	0.2 ± 0.4	**<0.001**
PLT [10^3^/μL] (140–440)	243 ± 90	234 ± 83	252 ± 81	0.144
LAC [mmol/L] (0.5–1.6)	1.2 ± 1.1	1.0 ± 0.8	1.6 ± 1.6	**0.001**
Glucose [mmol/L] (70–99)	156 ± 60	137 ± 48	169 ± 69	**<0.001**
Hb [G/dL] (14–18)	13.8 ± 2.1	13.5 ± 2.1	14.1 ± 2.1	0.057
Ht [%] (40–54)	40.8 ± 5.8	40.6 ± 6.1	41.1 ± 5.8	0.119
Na [mmol/L] (136–146)	138 ± 5	138 ± 4	138 ± 6	0.172
K [mmol/L] (3.5–5.1)	3.7 ± 0.8	3.8 ± 0.7	3.6 ± 0.7	0.753
Albumin [G/L] (35–52)	34 ± 6	35 ± 5	31 ± 7	**<0.001**

Abbreviations: CRP, C-reactive protein; WBC, white blood cell; PCT, procalcitonin; PLT, platelets; LAC, lactate; Hb, haemoglobin; Ht, haematocrit; Na, sodium; K, potassium; *p*, *p*-value; significant differences are marked in bold.

**Table 3:** ROC curve statistics for poor outcome predictors.

Parameter	Threshold Value	Z	*p*	AUC	SE	SPE
Biomarkers:						
CRP/albumin	0.167	2.09	**0.037**	0.59	0.51	0.73
D-dimer	1.62	5.57	**<0.001**	0.72	0.81	0.55
PCT	0.08	3.79	**<0.001**	0.67	0.69	0.62
LAC	1.3	3.38	**<0.001**	0.67	0.64	0.67
Glucose	153	6,63	**<0.001**	0.75	0.67	0.72
Albumin	33	5.79	**<0.001**	0.73	0.68	0.72
Covariates:						
Age	66	2.90	**0.004**	0.62	0.38	0.86
Apache II scale	17	12.27	**<0.001**	0.86	0.76	0.86
GCS scale	12	11.60	**<0.001**	0.84	0.82	0.73
FOUR scale	15	10.33	**<0.001**	0.83	0.88	0.66
H-H scale	4	10.69	**<0.001**	0.83	0.72	0.79
mFisher scale	4	7.19	**<0.001**	0.77	0.83	0.66
WFNS scale	4	10.36	**<0.001**	0.83	0.75	0.77
HIJDRA score	19	5.29	**<0.001**	0.71	0.67	0.68
SEBES	3	4.26	**<0.001**	0.67	0.56	0.73

Abbreviations: CRP, C-reactive protein; PCT, procalcitonin; LAC, lactate; Apache II, Acute Physiology and Chronic Health Evaluation; GCS, Glasgow Coma Scale; FOUR, Full Outline of UnResponsiveness scale; H-H, Hunt and Hess scale; mFisher, modified Fisher scale; WFNS grade, World Federation of Neurosurgical Societies grade; SEBES, Subarachnoid Haemorrhage Early Brain Edema Score; Z, value of Z-test statistics; *p*, *p*-value; AUC, area under the curve; SE, sensitivity; SPE, specificity; significant statistics are marked in bold; to determine the discriminative value of the biomarkers, receiver operating curves (ROC) were utilized, and the threshold was determined using the Youden index.

**Table 4:** Multivariate logistic regression analysis to predict poor outcome and delayed cerebral ischaemia (DCI) in a total cohort of aneurysmal subarachnoid haemorrhage (aSAH) patients.

Poor Outcome						
Factor	B	SE	W	*p*	OR	95% CI
SEBES	0.32	0.15	4.42	**0.035**	1.38	1.02–1.85
Apache II scale	0.18	0.04	19.31	**<0.001**	1.20	1.10–1.30
mFisher scale	1.23	0.36	11.72	**0.001**	3.43	1.70–7.00
albumin	−0.19	0.05	11.97	**0.001**	0.83	0.74–0.92
DCI development						
Factor	B	SE	W	*p*	OR	95% CI
Apache II scale	0.05	0.02	5.78	**0.016**	1.05	1.00–1.09
albumin	−0.11	0.04	10.53	**0.001**	0.88	0.82–0.95

Abbreviations: B, unstandardized coefficient; SE, standard error for the unstandardized beta, W, Wald statistic; *p*, *p* value; OR, odds ratio; 95% CI, 95% confidence interval; SEBES, Subarachnoid Haemorrhage Early Brain Edema Score; Apache II, Acute Physiology and Chronic Health Evaluation; mFisher, modified Fisher scale; significant differences are marked in bold.

## Data Availability

The data presented in this study are available on request from the corresponding author. The data are not publicly available due to privacy restrictions.

## Supplementary Materials

The following supporting information can be downloaded at: https://www.mdpi.com/article/10.3390/jcm12175614/s1, Figure S1. ROC curve for prediction of poor outcome based on (A) covariates, (B) biomarkers. Abbreviations: Apache II, Acute Physiology and Chronic Health Evaluation II scale; GCS, Glasgow Coma Scale; H-H, Hunt and Hess scale; mFisher, modified Fisher scale; SEBES, Subarachnoid Hemorrhage Early Brain Edema; WFNS, World Federation of Neurosurgical Societies; CRP, C-reactive protein; PCT, procalcitonin; LAC, lactate; Figure S2. Kaplan–Meier probability of survival for patients with aneurysmal subarachnoid haemorrhage (aSAH) regarding the levels of (A) albumin, (B) D-dimer, (C) glucose, and (D) procalcitonin (PCT); Figure S3. Matrix of confusion for random forest model to predict (A) poor outcome and (B) delayed cerebral ischaemia (DCI); Table S1. Analysis of survival: Kaplan–Meier analysis for biomarkers; Table S2. Baseline clinical characteristics of patients with aneurysmal subarachnoid haemorrhage (aSAH) in subgroups regarding high Subarachnoid Hemorrhage Early Brain Edema (SEBES) (3–4) and low SEBES (1–2). Data are presented as median ± interquartile range or number of subjects (%).
