# Supplementary material for: The Role of Early Serum Biomarkers and Clinical Rating Scales in the Prediction of Delayed Cerebral Ischaemia and Short-Term Outcome after Aneurysmal Subarachnoid Haemorrhage: Single Centre Experience
Source: J Clin Med. 2023 Aug 28;12(17):5614. doi: 10.3390/jcm12175614 (PMC10488375; doi:10.3390/jcm12175614)
Supplement: Supplementary file 1 [file jcm-12-05614-s001.zip › jcm-2536312-supplementary.pdf]

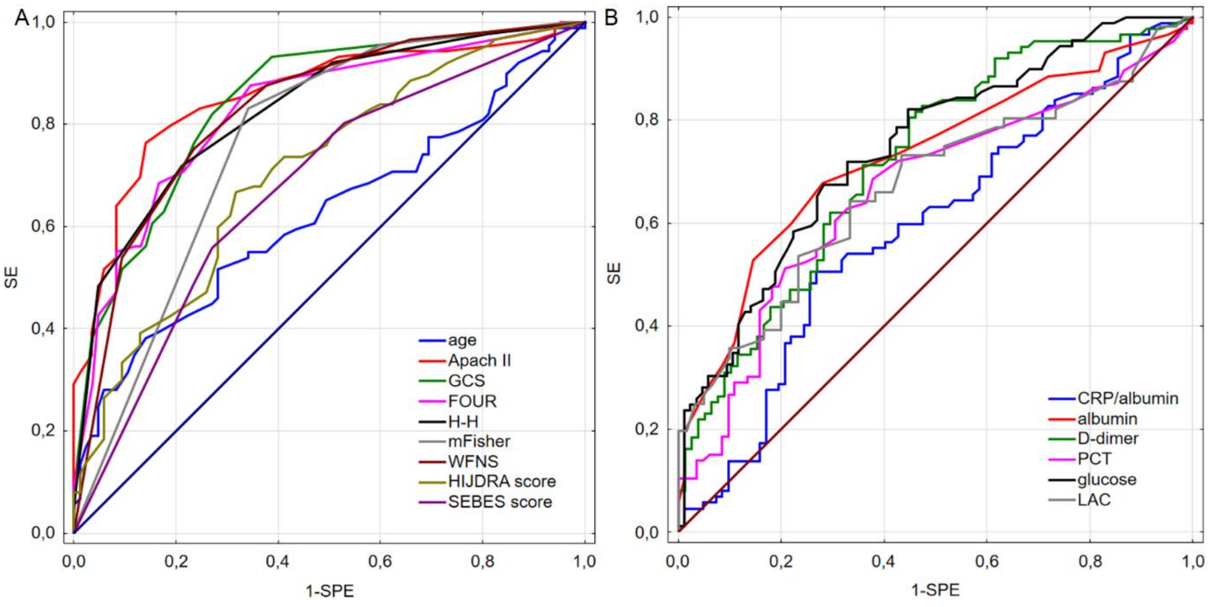

Figure S1. ROC curve for prediction of poor outcome based on (**A**) covariates, (**B**) biomarkers. Abbreviations: Apache II, Acute Physiology and Chronic Health Evaluation II scale; GCS, Glasgow Coma Scale; H-H, Hunt and Hess scale; mFisher, modified Fisher scale, SEBES, Subarachnoid Hemorrhage Early Brain Edema Score; WFNS, World Federation of Neurosurgical Societies; CRP, C-reactive protein; PCT, procalcitonin; LAC, lactate.

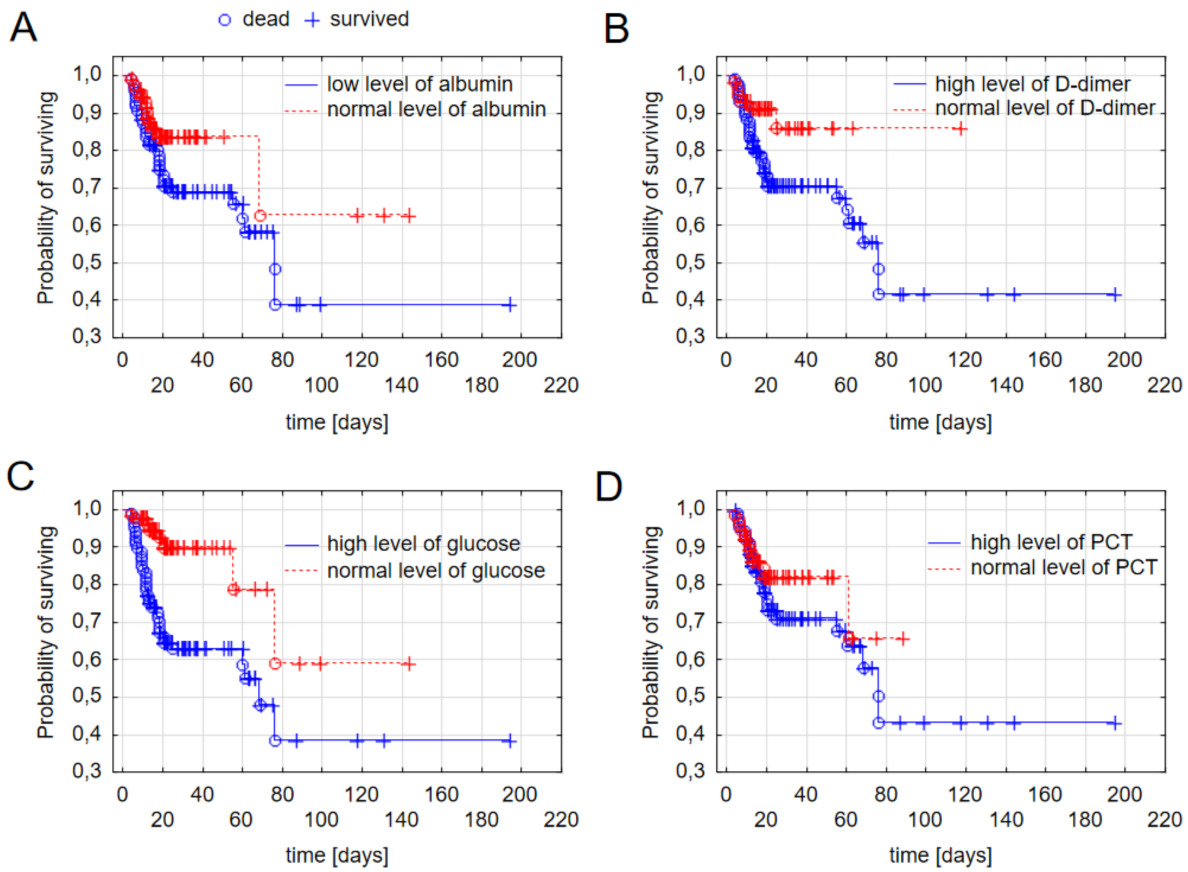

Figure S2. Probability of survival for patients with aneurysmal subarachnoid haemorrhage (aSAH) regarding the level of (**A**) albumin, (**B**) D-dimer, (**C**) glucose, and (**D**) procalcitonin (PCT).

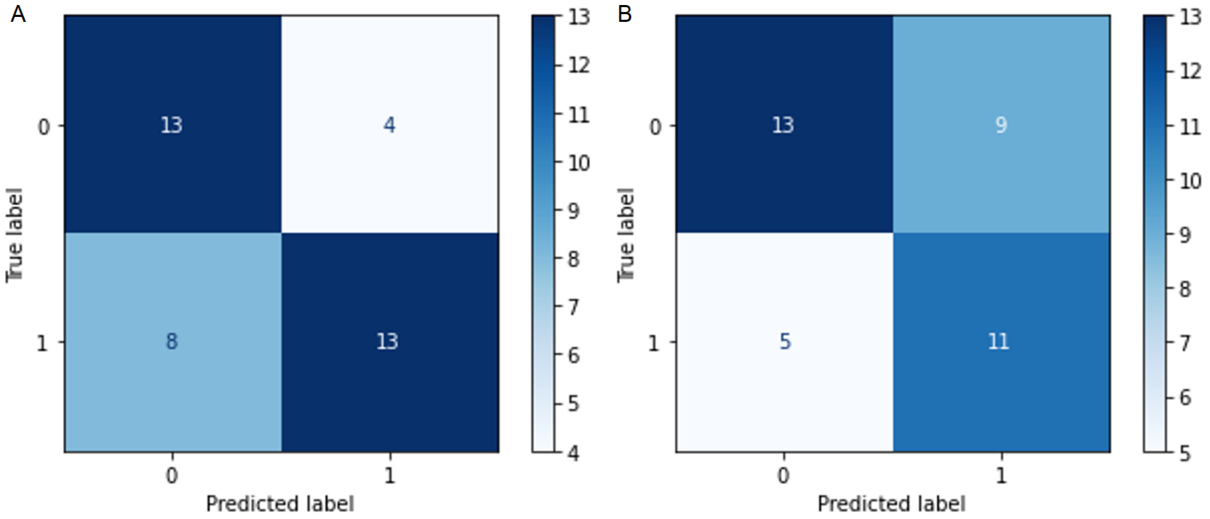

Figure S3. Matrix of confusion for random forest model to predict **A.** poor outcome, **B.** delayed cerebral ischemia (DCI).

Table S1. Analysis of surviving: Kaplan-Meier analysis for biomarkers.

Parameter	ROC-curve threshold	F Cox	p-value
CRP/albumin [a.u.]	0.17	1.24	0.249
Albumin [G/L]	33	2.53	**0.001**
D-dimer [ng/ml]	1.62	3.70	**< 0.001**
PCT [ng/ml]	0.08	1.88	**0.026**
LAC [mmol/L]	1.3	2.21	**0.029**
Glucose [mmol/L]	153	5.09	**< 0.001**

Abbreviations: CRP, C-reactive protein; PCT, procalcitonin; LAC, lactate; significant differences are marked in bold.

Table S2. Baseline clinical characteristics of patients with aneurysmal subarachnoid haemorrhage (aSAH) in subgroups regarding high Subarachnoid Hemorrhage Early Brain Edema Score (SEBES) (3-4) and low SEBES (1-2). Data are presented as median ± interquartile range or number of subjects (%).

Characteristic	SEBES 1-2<br>n = 103	SEBES 3-4<br>n = 71	p-value
Age	61 ± 18	51 ± 21	**<0.001**
Female	68 (66%)	34 (48%)	0.065
BMI	26.0 ± 6.8	25.7 ± 5.8	0.800
APACHE II	13 ± 11	20 ± 13	**<0.001**
GCS	13 ± 6	7 ± 8	**<0.001**
FOUR Score	16 ± 4	9 ± 9	**<0.001**
**Clinical assessment**			
H-H	3 ± 2	4 ± 2	**<0.001**
mFisher	4 ± 2	4 ± 1	0.075
HIJDRA	13 ± 22	22 ± 16	**<0.001**
WFNS	3 ± 2	4 ± 2	**<0.001**
CV in CTA	9 (9%)	12 (17%)	0.083
**Aneurysm treatment**			
Clipping	57 (55%)	36 (51%)	0.546
Coiling	42 (41%)	29 (41%)	0.992
EVD	39 (38%)	22 (31%)	0.093
Decompressive Craniectomy	14 (14%)	36 (51%)	**0.004**
**Outcome**			
ICU stay [days]	8 ± 7	13 ± 17	**< 0.001**
Hospital stay [days]	18 ±13	20 ± 34	0.381
Dead	20 (19%)	23 (33%)	0.052
**Neurologic complications**			
Cerebral infarction on follow-up CT	32 (31%)	34 (48%)	0.083
DCI	42 (41%)	37 (52%)	0.140
CV in TCD	50 (49%)	44 (70%)	0.073
**Systemic complications**			
Pneumonia	26 (25%)	34 (49%)	**0.002**
Inflammation of the urinary tract	23 (22%)	15 (22%)	0.850
Cardiac complications	4 (4%)	14 (20%)	**<0.001**
SIRS	28 (27%)	34 (48%)	**0.005**
Meningitis/ventriculitis	4 (4%)	3 (4%)	0.910
MOF	11 (11%)	13 (18%)	0.151
Neurogenic pulmonary oedema	20 (19%)	26 (36%)	**0.013**
Days on MV	3 ± 8	8 ± 12	**<0.001**

Biomarkers			
CRP [mg/L] (0-5)	6 ± 99	6 ± 105	0.367
CRP/albumin [a.u]	0.1 ± 0.4	0.1 ± 0.5	0.285
D-dimer [ng/mL] (0-0.5)	2.0 ± 3.1	2.6 ± 4.1	0.071
WBC [G/L] (4-10 10^3)	12.8 ± 6.3	14.0 ± 7.3	0.099
PCT [ng/mL] (0-0.05)	0.07 ± 0.14	0.15± 0.44	**<0.001**
PLT [10^3/uL] (140-440)	240 ± 86	250 ± 89	0.177
Lactate [mmol/L] (0.5-1.6)	1.1 ± 1.0	1.5 ± 1.9	**0.021**
Glucose [mmol/L] (70-99)	148 ± 57	166 ± 68	**0.015**
Hb [G/dl] (14-18)	13.8 ± 1.9	13.7 ± 2.8	0.451
Ht [%] (40-54)	40.3 ± 5.2	41.1 ± 8.2	0.408
Na [mmol/L] (136-146)	138 ± 5	138 ± 6	0.944
K [mmol/L] (3.5-5.1)	3.7 ± 0.7	3.6 ± 0.8	0.596
Albumin [G/L] (35-52)	34 ± 6	33 ± 9	0.100

Abbreviations: APACHE, Acute Physiology and Chronic Health Evaluation; BMI, body mass index; CT, computed tomography; CTA, computed tomography angiography; CRP, C-reactive protein; CV, cerebral vasospasm; DCI, delayed cerebral ischemia; EVD, external ventricular drainage; GCS, Glasgow Coma Scale; FOUR, Full Outline of UnResponsiveness scale, Hb, haemoglobin; Ht, hematocrit; H-H, Hunt and Hess scale; ICU, intensive care unit; K, potassium; mFisher, modified Fisher scale; MV, mechanical ventilation; MOF, multiple organ failure; Na, sodium; PCT, procalcitonin; PLT, platelets; SEBES, Subarachnoid Haemorrhage Early Brain Edema Score; SIRS, systemic inflammatory response syndrome; TCD, transcranial Doppler ultrasonography; WBC, white blood cells; WFNS grade, World Federation of Neurosurgical Societies grade; significant differences are marked in bold.
